# Is Repeat Transurethral Resection Always Needed in High-Grade T1 Bladder Cancer?

**DOI:** 10.3389/fonc.2019.00465

**Published:** 2019-06-04

**Authors:** Beppe Calò, Marco Chirico, Francesca Fortunato, Francesca Sanguedolce, Emanuel Carvalho-Dias, Riccardo Autorino, Giuseppe Carrieri, Luigi Cormio

**Affiliations:** ^1^Urology and Renal Transplantation Unit, Department of Medical and Surgical Sciences, University of Foggia, Foggia, Italy; ^2^Department of Epidemiology, University of Foggia, Foggia, Italy; ^3^Section of Pathology, Department of Clinical and Experimental Medicine, University Hospital of Foggia, Foggia, Italy; ^4^Urology Department, Hospital de Braga ICVS, University of Minho, Braga, Portugal; ^5^Division of Urology, Department of Surgery, VCU Health, Richmond, VA, United States

**Keywords:** non-muscle-invasive bladder cancer, second transurethral resection, Bacillus Calmette-Guerin, T1, high-grade

## Abstract

Re-staging transurethral resection, the so-called repeat TUR (Re-TUR), is mandatory in case of incomplete first transurethral resection of bladder tumor (TURBT). In completely resected high grade T1 tumors, Re-TUR is recommended but question remains whether it provides advantages in terms of recurrence-free survival (RFS), progression-free survival (PFS), and cancer specific survival (CSS). The present study aimed to determine whether Re-TUR improves such outcomes in patients with completely resected high-grade T1 bladder cancer (BC). We queried our prospectively maintained database to identify patients with completely resected high-grade T1 BC who underwent (Group A) or not (Group B) Re-TUR before starting intravesical instillations of Bacillus Calmette-Guerin (BCG). The impact of Re-TUR as well as of other tested variables on RFS, PFS, and CSS was tested by Kaplan-Meier method and Log-rank testing. A total of 118 patients underwent Re-TUR, which pointed out no BC in 61 (51.7%), NMIBC in 54 (45.8%) and pT2 disease in 3 (2.5%). The 3 patients with pT2 disease underwent cystectomy, whereas all others were offered BCG treatment. Forty-two patients refused BCG treatment while 2 did not complete it; therefore, Group A (Re-TUR before BCG treatment) consisted of 71 patients whereas Group B consisted of 40 patients who refused Re-TUR but completed BCG treatment. Mean follow-up was 60 months (range 12-142). Kaplan-Meier curves and Log-rank testing showed no difference in RFS, PFS and CSS between patients who had (Group A) or had not (Group B) Re-TUR before starting BCG treatment. Our findings suggest that a Re-TUR in patients with a completely resected high-grade T1 BC does not translate into a better oncological outcome. Given its impact on both patients and healthcare system, the need for Re-TUR in completely resected high grade T1 BC should be further investigated into the framework of a randomized study.

## Introduction

The first and mainstay approach in the diagnosis and treatment of bladder cancer (BC) is transurethral resection of the bladder tumor (TURBT). Complete resection is crucial in the management of patients with high-risk non-muscle-invasive bladder cancer (NMIBC) (1). A restaging transurethral resection, the so-called repeat TUR (Re-TUR), which is mandatory in case of incomplete first resection, is currently recommended in high-grade T1 tumors, either primary or recurrent, even in case of a complete first TURBT (2).

In one of his first study about this issue Herr claimed that Re-TUR was especially appropriate for patients with T1 tumors to confirm complete resection and to detect muscle invasion (3). In the following years it has been shown that this can provide additional pathologic information, as residual Ta/T1 lesions are found in 33–55% of patients and muscle-invasive (T2) disease can be detected in 10–25% of patients (4–7). Question however remains whether Re-TUR provides advantages in terms of recurrence-free survival (RFS), progression-free survival (PFS), and cancer specific survival (CSS).

The first and only randomized controlled trial (RCT) addressing this issue (8) pointed out that, in patients with T1 BC, Re-TUR provided a significant benefit in RFS and PFS but not in CSS. It is worth mentioning that almost half of patients had a T1 low-grade disease and that patients received intravesical instillations of Mitomycin-C rather than of Bacillus Calmette-Guerin (BCG), which should be the standard treatment for high-risk NMIBC (8, 9). The largest retrospective study testing the role of Re-TUR in a homogeneous population of patients with high-grade T1 BC treated with intravesical BCG (10) conversely pointed out that, in case of complete resection, Re-TUR did not improve RFS, PFS, and CSS. These findings would question the need for such additional surgical procedure, considering its patients and healthcare burdens. The present study aimed to determine the effect of Re-TUR on RFS, PFS, and CSS in a homogeneous population of patients with high-grade T1 BC treated with BCG.

## Materials and Methods

Our prospectively maintained NMIBC database was queried to identify patients with high-grade T1 BC, either primary or recurrent, who underwent Re-TUR (Group A) or did not undergo Re-TUR (Group B) before receiving adjuvant intravesical instillations of BCG. The study was approved by the Internal Review Board, Nephro-Urological Department, Foggia University Hospital, Foggia, Italy. All subjects gave written informed consent in accordance with the Declaration of Helsinki.

Study inclusion criteria were: (i) complete resection, i.e., no visible tumor left behind and bladder muscle clearly identifiable by pathologist and free of disease; (ii) having completed the BCG treatment (induction by one instillation a week for 6 consecutive weeks and maintenance by one instillation a month for 12 months); (iii) having undergone bladder biopsies (random and/or visible lesions) 4–8 weeks after having completed the BCG induction cycle. Patients with incomplete clinical data were excluded.

Re-TUR involved resection of any visible tumor, deep resection of previously resected areas, and random bladder biopsies. As mentioned above, all patients underwent urine cytology and bladder biopsies (random and/or visible lesions) 4–8 weeks after having completed the BCG induction cycle, as we aimed to evaluate the role of this diagnostic pathway in assessing the results of BCG treatment.

Follow-up consisted of urine cytology and cystoscopy every 3 months for the first two years, every 6 months for the third year, and then yearly. A CT urogram was also performed at initial diagnosis and then every year to rule out upper tract or metastatic disease. Tumor recurrence was defined as pathological evidence of disease at bladder biopsy or TURBT, whereas tumor progression was defined as pathological shift to muscle invasive disease at bladder biopsy or TURBT or evidence of metastatic disease.

Two senior pathologists unaware of clinical data reviewed all specimens including agreement with the latest WHO Classification of Tumors of the Urinary System and Male Genital Organs (11) and the 2010 TNM staging system (12). The study was approved by the Internal Review Board.

### Statistical Analysis

Continuous data are reported as means ± standard deviations (SD) or median values as appropriate.

Those with normal distribution according to the Skewness and Kurtosis test were compared by Student's *t*-test for paired or unpaired data, whereas those with a non-parametric distribution were compared by the Mann-Whitney *U*-test. Differences in rates were compared by the chi-square test or the Fisher's exact test. Univariate survival analysis was carried out using the Kaplan-Meier method, with differences among groups being tested for significance using the Log-rank test. Significance was set at *p* < 0.05. Statistical analysis was carried out using the MedCalc 16.8 Software (MedCalc, Ostend, Belgium) and STATA SE 14.

## Results

**Figure 1:**
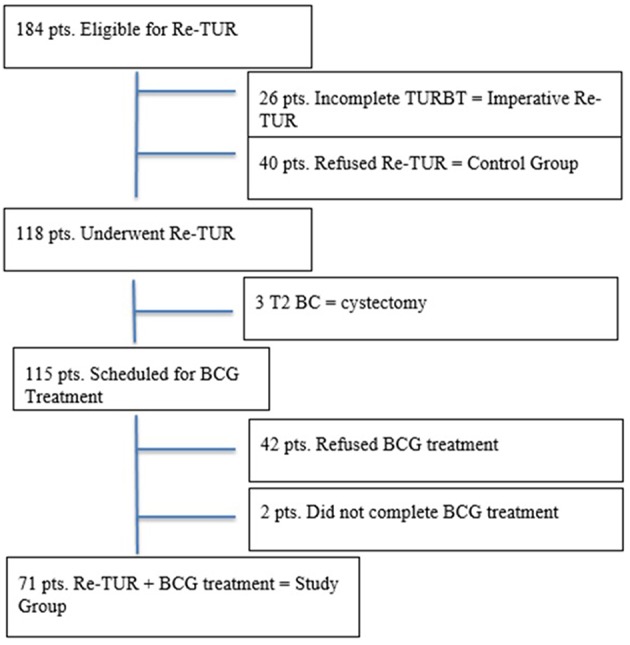
Flowchart patients selection.

From January 2005 to January 2017, a total of 184 patients with high-grade T1 BC were offered Re-TUR before being scheduled for BCG treatment (Figure 1). Forty patients refused Re-TUR but completed BCG treatment; they represent controls (Group B). Twenty-six patients were excluded from this study as Re-TUR was carried out because of incomplete resection or absence of detrusor muscle in the specimen. In the remaining 118 patients, Re-TUR (Table 1) pointed out no BC in 61 (51.7%), NMIBC in 54 (45.8%), and pT2 disease in 3 (2.5%). The three patients with pT2 disease underwent cystectomy, whereas all others were offered BCG treatment; forty-two patients refused it while two did not complete it, thus leaving 71 patients who had Re-TUR and completed BCG treatment (Group A).

**Table 1:** Results of Re-TUR in completely resected high grade T1 BC.

T2, n (%)	3 (2.5)
T1 HG, n (%)	19 (16.1)
Ta HG, n (%)	10 (8.5)
Ta HG + CIS, n (%)	1 (0.8)
Ta LG, n (%)	7 (5.9)
CIS, n (%)	17 (14.4)
No BC	61 (51.7)

*HG, high-grade; LG, low grade; CIS, Carcinoma in situ*.

Table 2 compares the characteristics of patients in Group A and B showing that, apart from tumor multifocality being significantly more common in Group A, the two groups had similar characteristics.

**Table 2:** Patients' characteristics.

	**Group A = 71 pts**	**Group B = 40 pts**	***p-value***
Mean Age (y)	67.9 ± 9.6	69.7 ± 11.2	0.2004
Female Gender, *n* (%)	18%	8.1%	0.1172
Primary, *n* (%)	56 (78.9)	26 (65)	0.1866
Multifocal, *n* (%)	51 (71.8)	11 (28.2)	0.0001
Concomitant CIS, *n* (%)	3 (4.2)	1 (2.5)	0.5365
Diameter BC > 30 mm, *n* (%)	27 (38)	15 (37.5)	0.8990

*CIS, Carcinoma in situ*.

Bladder biopsies after BCG treatment showed was no difference in the tumor recurrence rate between Group A and B (12.6 vs. 15%, respectively, *p* = 0.765). In particular bladder biopsies after BCG treatment showed, in Group A, 2 high-grade T1 with concomitant CIS, 3 de novo CIS, and 4 low grade Ta BC. The two patients with recurrent high-grade T1 and concomitant CIS underwent cystectomy; those with de novo CIS had a second BCG induction cycle while the others had BCG maintenance. In Group B bladder biopsies after BCG treatment showed 1 CIS, 3 high-grade Ta, 1 high-grade T1, and 1 high-grade T2 BC. Patients with no tumor recurrence or with recurrent low grade disease were scheduled for BCG maintenance; the patient with T2 disease underwent cystectomy. The remaining patients with recurrent high-grade disease or CIS had a second BCG induction cycle.

The mean follow-up of the 108 patients (69 in Group A and 39 in Group B) who remained on “conservative” treatment was 60 months (range 12–142). Recurrence occurred in 33 patients, 19 (27.5%) in Group A, and 14 (35.8%) in Group B. Progression occurred in 17 patients, 9 (13%) in Group A, and 8 (20.5%) in group B. Eight progressions occurred after disease recurrence and consisted of local disease in 5, local disease + liver metastases in 1 and local disease + lung metastases in 2; they were 4 (5.8%) in Group A and 4 (10.3%) in Group B. Nine patients presented direct disease progression (7 local diseases and 2 associated to multiple pulmonary metastases); they were 5 (7.2%) in Group A and 4 (10.3%) in Group B. Of the 17 patients who progressed, 11 underwent cystectomy; 9 patients died because of their BC (Table 3).

**Table 3:** Oncological outcomes.

	**All (108 pts)**	**Group A (69 pts)**	**Group B (39 pts)**	*p*-value^*^
Recurrence, *n* (%)	33 (30.5)	19 (27.5)	14 (35.8)	0.346
Progression, *n* (%)	17 (15.7)	9 (13)	8 (20.5)	0.199
Cancer-related death, *n* (%)	9 (8.3)	3 (4.3)	6 (15.3)	0.046

* *Chi-square test*.

**Figure 2:**
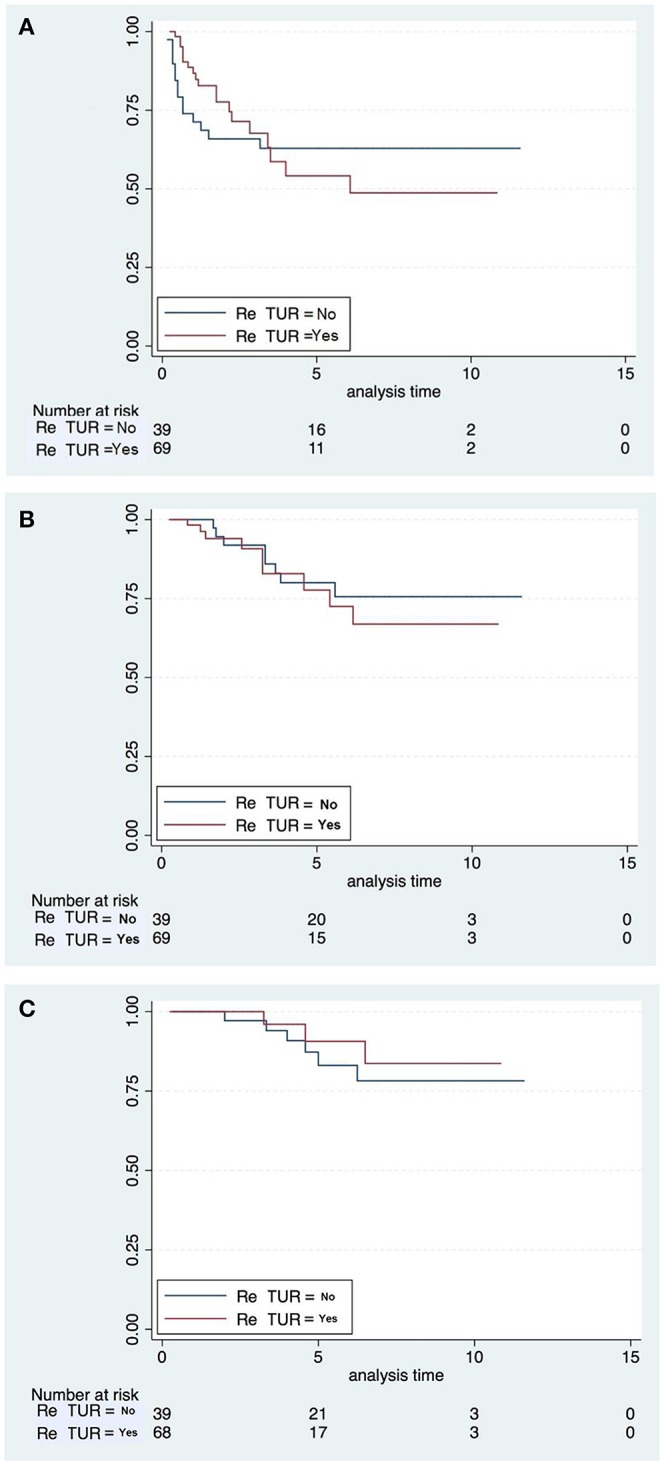
Kaplan-Meier plots for RFS **(A)**, PFS **(B)**, and CSS **(C)**.

Kaplan-Meier curves (Figures 2A–C) and Log-rank testing showed no difference in RFS, PFS and CSS between patients who had or not Re-TUR. Indeed, also the other tested variables had no impact on RFS, PFS, and CSS, as Kaplan-Meier curves and Log-rank testing showed no difference for males vs. females, primary vs. recurrent tumors, single vs. multiple tumors, tumor size < 3 cm vs.>3 m, and presence vs. absence of CIS (Table 4).

**Table 4:** Univariate analysis^*^ of impact of tested variables on recurrence-free survival (RFS), progression-free survival (PFS) and cancer-specific survival (CSS).

	**RFS**	**PFS**	**CSS**
Male vs. Female	0.137	0.118	0.293
Primary vs. Recurrent	0.785	0.301	0.516
Single vs. Multifocal	0.264	0.536	0.231
Size < 3 cm vs. >3 cm	0.416	0.211	0.734
no CIS vs. CIS	0.209	0.475	0.640
No ReTUR vs. Re-TUR	0.854	0.586	0.489

*RFS, Recurrence Free Survival; PFS, Progression Free Survival; CSS, Cancer Specific Survival; Pts: patients*.

* *Kaplan-Meier method and Log-rank testing. Data are expressed as p-values*.

## Discussion

Several findings from our analysis are worth discussing within the framework of current literature.

About 46% of our patients undergoing Re-TUR after a completely resected high-grade T1 BC were found to have NMIBC, while only 2.5% were found to have T2 disease. While the rate on NMIBC is in line with previous studies (1, 5, 6), the rate of T2 disease is lower and consistent with that (2.5%) yielded by bladder biopsies after BCG treatment in those who did not undergo Re-TUR. Taken together, these figures somehow speak for appropriate depth of our initial TURBT.

Even more interesting, bladder biopsies after BCG treatment showed no significant difference in the tumor recurrence rate between group A and B. Since it is reasonable to assume that Re-TUR would have yielded an almost 45.8% rate of recurrent/residual disease also in Group B, the discrepancy in the rate of recurrent/residual disease between Re-TUR (45.8%) in group A and post-BCG bladder biopsies in group B (15%) would speak for the ability of BCG induction cycle to eradicate limited recurrent/residual disease in many cases. BCG treatment has been shown to modulate the urinary expression of multiple molecules regulating proliferative and angiogenic activity (13). Such cytotoxic, pro-apoptotic, and hypoxic effects of BCG would explain its ability to eradicate at least minimal residual tumors after the initial TURBT, thus explaining our two groups having similar rate of BC at bladder biopsies after the BCG induction cycle. Indeed, Oosterlinck et al. already demonstrated that more than 50% of exophytic Ta-T1 tumors (< 10 mm) regressed after 6 weeks of BCG treatment (14), and Mack et al. (15) showed that BCG is able to ablate residual disease in a marker lesion study.

The most relevant question however remains whether Re-TUR improves RFS, PFS and CSS. The first RCT addressing this issue was carried out by Divrik et al. (16) who tested the role of Re-TUR in patients with T1 BC scheduled for adjuvant treatment with intravesical Mitomycin-C after initial TURBT. Re-TUR pointed out NMIBC in 33.3% of cases and upstage to T2 disease in 7.6% of cases.

Of note, Kaplan-Meier curves indicated a significant benefit in RFS and PFS but not in CSS for patients having undergone Re-TUR. Multivariable analysis stated that tumor number, tumor grade and Re-TUR were all significant independent predictors of disease recurrence, whereas tumor size and Re-TUR were significant independent predictors of disease progression. Though such findings would suggest Re-TUR to improve RFS and PFS, it is worth mentioning that this study included both low-grade and high-grade T1.

In a large retrospective study, Sfakianos et al. (17) compared RFS and PFS of patients they treated with intravesical BCG for high-risk NMIBC who had (*n* = 894) or had not (*n* = 127) undergone Re-TUR. Kaplan-Meier curves showed that Re-TUR provided a significant advantage in both RFS and PFS; multivariable analysis established that Re-TUR was the only significant predictor of recurrence at 5 years. Unfortunately, also this study suffers the biases of including tumors of different stages (Ta and T1) and grades (high-grade and low-grade), as well as not having evaluated at all the impact of prognostic factors on disease outcome.

The most interesting study defining the role of Re-TUR in a large yet homogeneous population of patients with high-grade T1 BC treated with intravesical BCG was carried out by Gontero et al. (10) Re-TUR, which was carried out in 935 (38%) of the 2451 patients, had a positive impact on RFS, PFS, and CSS only if muscle was not present in the primary TURBT specimen. However, adjusting for the most important prognostic factors, Re-TUR in the absence of muscle had a borderline significant effect on RFS, PFS, and CSS, whereas Re-TUR in the presence of muscle in the primary TURBT specimen did not improve the outcome for any of these endpoints.

In agreement with Gontero's findings, we found that, in a homogeneous population of completely resected high-grade T1 BCs treated with BCG, Re-TUR did not improve RFS, PFS, and CSS.

Whether or not this finding is due to the efficacy of BCG treatment remains speculative.

Independently on such speculation, our data further support Gontero's conclusions that Re-TUR can be avoided in high-grade T1 BC providing it has been completely resected and the muscle is clearly visible and free of disease. The potential clinical relevance of such findings is obvious, as avoiding unnecessary Re-TURs means a significant reduction of both patient discomfort for the additional surgical procedure and healthcare costs.

The main limitations of our study was the absence of randomization, as the decision not to perform Re-TUR was a patient's informed choice. However, data were prospectively collected, and the two study groups had similar characteristics. Another study limitation was the relatively small sample size, but a single center study focusing on high-grade T1 tumors only should, however, guarantee for consistency in patients' population and study methodology.

## Conclusions

Our study findings question the role of repeat TUR in case of a completely resected (muscle available and disease-free) high-grade T1 BC as this procedure might not translate into a better oncological outcome. In view of patient discomfort and healthcare costs associated with Re-TUR, the role of this procedure should be further investigated, ideally into the framework of a randomized study.

## Data Availability

The datasets generated for this study are available on request to the corresponding author.

## Author Contributions

BC, MC, FF, FS, EC-D, RA, GC, and LC participated in the study design. BC and MC performed the experiment. BC, MC, FF, FS, EC-D, RA, GC, and LC were involved in data collection and data interpretation. BC, MC, and FF participated in the statistical analyses. BC, RA, and LC wrote the manuscript. All authors read and approved the final manuscript.

### Conflict of Interest Statement

The authors declare that the research was conducted in the absence of any commercial or financial relationships that could be construed as a potential conflict of interest.
